# Primary health care seeking behaviour of people with physical disabilities in Bangladesh: a cross-sectional study

**DOI:** 10.1186/s13690-018-0293-1

**Published:** 2018-09-03

**Authors:** Jhalok Ronjan Talukdar, Ilias Mahmud, Sabina Faiz Rashid

**Affiliations:** 1 BRAC James P Grant School of Public Health, BRAC University, Dhaka, Bangladesh; 2 Department of Health Research Methods, Evidence, and Impact, McMaster University, Hamilton, ON Canada; 3 College of Public Health & Health Informatics, Qassim University, Bukayriah, Qassim Kingdom of Saudi Arabia

**Keywords:** Disability, Physical disability, Person with physical disabilities, Health care seeking behaviour, Factors of primary health care seeking behaviour

## Abstract

**Background:**

People with disabilities constitute about 10% of the total population of Bangladesh. They are more likely to experience poor health than those without disabilities. However, there is a lack of evidence on their primary health care (PHC) seeking behaviour for their general illness. The aim of this study was to understand the PHC seeking behaviour of people with physical disabilities (PWPDs), and to investigate the determinants of such behaviours.

**Methods:**

We surveyed 282 PWPDs, aged ≥18 years, using a structured questionnaire. Participants were recruited from the out-patient department of a rehabilitation centre in Dhaka between November and December 2014. We explored PHC seeking behaviour using frequency distribution. We performed logistic regression to investigate the factors that determined their PHC seeking behaviours for general illness. In our logistic regression model, the outcome variable was whether PWPDs received treatment from a formal health care provider. The predictors were socio-demographic characteristics and clinical characteristics such as type of impairment and type of illness experienced.

**Results:**

Among 282 participants, 85% suffered from general illness in the past 6 months. The participants in the higher age group, for example, age group 31–45 years (OR = 3.9, [95% CI 1.2 to 13.4]), 46–59 years (OR = 13.6, [95% CI 2.9 to 63.7) and 60+ years (OR = 12.5, [95% CI 1.7 to 93.0]) were more likely to seek treatment from formal health care providers than the age group 18–30 years. The educational attainment of the primary income earning family member (OR = 3.2, [CI 1.1 to 9.6]), religion (OR = 0.3, [95% CI 0.1 to 0.98]) and mobility aid used (OR = 4.0, [95% CI 1.2 to 13]) were determinants for seeking health care from a formal health care provider. Moreover, the type of illness suffered by participant was a strong predictor of their decision to seek treatment from a formal health care provider. The participants who suffered from urinary tract infections (OR = 10.3, [95% CI 2.3 to 46.6]), ulcers (OR = 13.1, [95% CI 2.11 to 79.3]) and pain (OR = 3.6, [95% CI 1.4 to 9.4]) were more likely to seek treatment from formal health care provider than who suffered from fever.

**Conclusions:**

Age, religion, earning member’s education, type of mobility aids used and type of illness suffered were explicative determinants of PHC seeking behaviour of PWPDs. The results suggest that these factors should be considered when devising interventions for this population. Moreover, accessibility, quality of care and expertise of the providers in treating disabled people were among the other factors reported by PWPDs which influence their decision to seek health care. In order to provide inclusive health services, primary health centres need to consider these determinants.

## Background

Disability is the negative outcome of a complex interaction between an individual’s health conditions and his/her personal, environmental and social contexts [1, 2]. This negative interaction results in limitations in performing activities and restrictions in participation in life situations. We defined physical disability as an umbrella term of physical impairment, activity limitations and participation restrictions [1, 3].

Globally more than one billion people are living with some form of disability, and around 110 to 190 million adults are experiencing difficulties in performing everyday activities and participating in life situations [4]. Currently, we are observing an increase in the size of the aging population and a shift of in the global burden of diseases from communicable to non-communicable diseases. As a result, the number of people with disabilities is increasing globally [4].

Although the number of people with physical disabilities (PWPDs) is undoubtedly large and increasing globally [4], there is a lack of primary health care services sensitive to the special needs of PWPDs and they are rarely targeted by preventive health care services or health promotion activities tailored to their needs [5]. A number of factors such as prohibitive cost and limited availability of health care services, physical barriers to visiting health care facilities, inadequate knowledge and skills on the part of health care providers and perceived discrimination have a negative impact on PWPDs and prevent them from seeking health care [6–8]. Socio-demographic characteristics such as age, socio-economic status, education and place of residence also have a strong influence on health care seeking behaviour of PWPDs [8, 9]. Moreover, perceived discrimination also play an important role on health care seeking behaviour of PWPDs [10].

People with disabilities are more likely to suffer from health conditions requiring primary health care services than people without disabilities. Bangladesh has a high prevalence of disability. The Bangladesh Bureau of Statistics (BBS) [11] estimated the prevalence of disability in Bangladesh at 9.07% for male and 10% for female. A study by Titumir and Hossain [12] found that among the individuals living with disability in Bangladesh, 27.8% have physical impairment and 10.7% have multiple impairments. Therefore, the number of people with physical disabilities is believed to be very high, given the country has around 158 million people [13]. However, there is a lack of research evidence related to primary health care seeking behaviour of PWPDs in Bangladesh. Few studies [14–17] were conducted in Bangladesh to explore the health care seeking behaviour of PWPDs for the treatment and rehabilitation of their disabling health condition but no such studies were conducted related to the primary health care seeking behaviour of PWPDs.

Knowledge of health care seeking behaviour is helpful in identifying available health services and assessing utilization of these services by the community. This knowledge can also be used to promote positive changes in health care seeking behaviour of the target population [18]. Improving access to quality health care can prevent the decline in income frequently caused by ill health [19]. To ensure the effectiveness of health interventions for PWPDs, it is necessary to ensure the participation of PWPDs. In this regard it is important to understand the needs and priorities of PWPDs related to their general health. Proper understanding of health care seeking behaviour for general illness and the factors determining health care seeking behaviour will help policy makers to design effective public health interventions in a way that will ensure participation of PWPDs. Moreover, this information will help policy makers to minimize the barriers to health care seeking behaviours. For example, if accessibility in health care settings is a major barrier to seeking health care by PWPDs in Bangladesh, then policy makers might try to understand what the current accessibility options are and what could be done to improve accessibility for PWPDs. If the issue is a lack of access to certain medical and rehabilitation professionals in the health care settings, then they might try to understand what specialized medical and rehabilitation professionals are required by PWPDs and make them available in health care settings. Understanding these barriers will help policy makers to take the proper measures so that PWPDs do not perceive or experience barriers to seeking health care.

The objective of the study was to describe the prevalence of primary health care seeking by PWPDs and to identify the determinants of the health care seeking behaviour of PWPDs for their general illness. This study will fill a gap in research regarding the health care seeking behaviour of PWPDs for their general illness, apart from disabling health condition. It will inform the development of primary health care interventions for PWPDs in Bangladesh.

## Methods

### Study design

This was a cross-sectional study carried out at the Centre for the Rehabilitation of the Paralysed (CRP), Dhaka, Bangladesh.

### Study setting

CRP is a specialized treatment and rehabilitation centre for PWPDs in Bangladesh. It is located approximately 24 km northwest of Dhaka. CRP was selected as our study site as it is the largest tertiary care facility for PWPDs in Bangladesh. PWPDs from all over Bangladesh visit this centre for treatment and rehabilitation (http://www.crp-bangladesh.org/).

### Conceptual model

This study was guided by an adaptation of Anderson’s behavioural model of health services (as cited in [19]) which organizes all the factors that predict health care seeking behaviour into three broad categories - need factors, enabling factors and predisposing factors. ‘Need factors’ include the individual’s perception of the severity of their health problems. ‘Enabling factors’ include factors such as affordability and availability of services and social network support. ‘Predisposing factors’ include individual characteristics such as age, sex, education, occupation, attitudes and beliefs regarding health care services and use of these services. We operationalized ‘need’ as the PWPDs’ perceptions of the severity of their health problems which can be dealt at the primary health care other than the condition which contributed in physical disability. We considered the financial condition of the participants to be ‘enabling factors’. We considered socio-demographic characteristics and individuals’ perceptions regarding health care seeking behaviour for a specific illness to be ‘predisposing factors’.

### Study participants

The participants of the study were all PWPDs age 18 years or older who were visiting the CRP between November and December 2014 for physiotherapy or occupational therapy for their disabling health condition and met our predefined inclusion criteria. We included those participants who were living in their community with their physical disability for at least the past 6 consecutive months. We excluded those PWPDs who experienced a medical emergency or needed inpatient rehabilitation while receiving care at CRP.

### Sampling technique

We employed a convenience sampling technique and included those PWPDs consecutively who sought treatment at the CRP during the study period.

### Data collection

We used a structured interviewer administered questionnaire for data collection. In order to ensure the reliability and validity of data, we developed the questionnaire based on literature review. Two disability rehabilitation professionals then reviewed the questionnaire. Finally, it went through field testing and was revised accordingly. The first author and three data collectors interviewed all respondents. They received a two-day training on administering the questionnaire and potential ethical issues led by the senior researchers.

### Dependent and independent variables

The independent variables for this study were participants’ age, gender, religion, educational attainment, occupation, individual and household monthly income, form of impairment, types of mobility aids used, area of residence and type of illness suffered during the past 6 months. In addition, relationship with key earning member, household key earning members’ educational attainment and occupation were used as independent variables.

Participants’ age was originally recorded in completed years, but later recoded into four groups: 18–30 years, 31–45 years, 46–59 years and 60 years or above. Educational attainment was classified into the following educational groups, based on the highest level completed: less than primary education, primary education, lower secondary education, upper secondary education and post-secondary or above. Occupation was classified into the following groups- elementary occupation (included agricultural, industrial and day labourers), government employees, non-government organization (NGO) or private employees (included clerical staff, mangers, teachers and professionals), students (studying in formal institutions), homemakers (individuals managing household/family activities) and unemployed. Participants were classified into the following three impairment groups: paraplegia or tetraplegia, hemiplegia, and others which included any conditions affected a single arm or leg. Income was reported in Bangladeshi Taka (BDT) which was converted to US dollar (USD) and respondents were divided into two income groups: below median income level and median income level or above. The dependent variable was whether the PWPDs received treatment from formal health care providers.

### Data analysis

Data were analysed using Stata/MP 13.0. Descriptive analyses were done to explore participants’ socio-demographic and disability profile, primary health care seeking behaviour and the factors that shaped these behaviours. Bivariate analysis was done to understand the factors associated with seeking health care from formal health care provider. The associations between dependent and independent variables were investigated using Pearson chi-square or Fisher’s exact tests.

Multivariate analysis was done to explain the health care seeking behaviour of PWPDs. In this case, a logistic regression model was developed to investigate the determinants for seeking treatment from a formal health care provider. This model is based on the purposeful selection of variables. In this case, besides the variables which demonstrated significant associations in bivariate analyses, some important variables were also added in the final model which were not significant in bivariate analyses but might be important if they were used with significant variables in the same model. Then specification error was checked to understand whether the model included the important independent variables and that none were excluded. The goodness-of-fit test suggested by Hosmer and Lemeshow was checked. The reported large *p*-values (*p* = 0.63) of the test were an indication that the data fit the model very well. Moreover, the multicollinearity of the final model was checked using variance inflation factor (VIF) and tolerance (which is 1/VIF). If VIF is greater than 10 or tolerance is less than 0.1 for any variable, then these variables need further investigation. However, for the final model the VIF for all variables was around 1 which was an indication that there was no multicollinearity problem in this model.

## Results

### Socio-demographic profile of the participants

In total, 282 participants were included in the data analysis. Among the 282 participants, over two fifths (43%) were 18 to 30 years old and majority currently married (63%). About 28% of the participants had only completed primary education and more than half (61%) of them did not have an economically productive occupation (Table 1).

**Table 1:** Socio-demographic profile of the participants, a cross-sectional study of primary health care seeking behaviour of adults with physical disabilities in Bangladesh, November–December 2014

Variables	Frequency (%)
Age (years)	
18–30	120 (43)
31–45	96 (34)
46–59	45 (16)
60–80	21 (7)
Sex	
Male	237 (84)
Female	45 (16)
Religion	
Muslim	259 (92)
Hindu	23(8)
Educational attainment	
Less than primary education	76 (27)
Primary education	78 (28)
Lower secondary education	43 (15)
Upper secondary education	43 (15)
Post-secondary or above	42 (15)
Occupation	
Elementary occupation	18 (6)
Government, NGO or private employee	37 (13)
Small business	54 (19)
Student	42 (15)
Homemaker	23 (8)
Unemployed	108 (38)
Mean monthly income (USD)	
Individual	77
Household (HH)	265
Form of impairment	
Paraplegia/tetraplegia	123 (44)
Hemiplegia	50 (18)
Others	109 (39)
Type of mobility aids used	
Wheel chair	138 (49)
Crutch or stick	89 (32)
No mobility aid	55 (19)
Area of residence	
Urban	168 (60)
Rural	114 (40)
Last illness suffered	
UTI	26 (11)
Sores/ulcers	16 (6)
Fever	82 (34)
Pain	52 (22)
Diarrhea	17 (7)
Others	47 (20)
Key earning member in the HH	
Participant	101(36)
Father or Mother	77 (27)
Brother or Sister	31 (11)
Wife	39 (14)
Others	34 (12)
HH key Earner’s educational attainment	
Less than primary education	98 (35)
Primary education	69 (24)
Lower secondary education	31 (11)
Upper secondary education	37 (13)
Post-secondary or above	47 (17)
HH key earner’s occupation	
Elementary occupation	45 (16)
Government, NGO or private employees	80 (28)
Business	109 (39)
Unemployed	48 (17)

In more than a third case (36%) the participant him/herself was the main earning member of the household. About 35% of the main earning member had not completed primary education and 83% of main earning members were engaged in an economically productive occupation. About 70% of the households had less than or equal to five members and more than half of them had monthly income of more than 145 USD. The majority (60%) of participants were living in urban areas.

### Physical impairment of participants

The most common impairment experienced by participants was paraplegia/tetraplegia (44%), and 49% of participants in this group used a wheelchair. About 89% of the participants had an acquired disability versus 11% who lived with a congenital disability. Most of the participants (57%) had lived with this disability for more than 2 years.

### Primary health care seeking behaviour of PWPDs

Of the 282 study participants, 239 suffered from different forms of general illness, apart from their disabling condition, that can be dealt in the primary health care centres in Bangladesh, in the last 6 months. The participants mostly suffered from fever, pain and infection (34, 22 and 17% respectively). Among those who experienced a general illness, 88% of them took some form of treatment. Of those participants who took treatment, 55% of them took it within one day. Some participants delayed treatment, that is they waited more than 3 days after onset of the symptoms to seek the treatment. The majority of these participants (35%) faced mobility related barriers to access treatment services because they needed help from others to visit the health care facilities.

More than two-thirds (69%) of the health care providers they consulted for a general illness, were formal health care providers. The reasons the participants revealed for consulting a formal health care provider over an informal health care provider included specialized (96%) and good quality (74%) care, which they felt they could only get from a formal health care provider. Easy access (63%) was another important factor that led them to seek treatment from a formal health care provider. Most participants visited a private clinic or hospital (32%), government hospital (30%) or NGO clinic or hospital (30%) to consult a formal health care provider.

Among the participants who sought treatment from an informal health care provider (31%), 17% of them obtained treatment from a pharmacy and 5% used self-care treatments. About 20% of the participants consulted both formal and informal health care providers, 39% of these participants mentioned that they took several different forms of treatment as they were desperate to get well quickly.

### Expectations that influence decisions to visit health facilities

The participants who took at least one form of treatment considered several issues before visiting a health care facility. Among the 239 participants who sought any form of treatment from any health facility, 82% of them prioritized choosing a health facility where they felt they would receive quality treatment. An equal number of participants (56% each) expected specialized care and good communication when visiting a health facility. These factors were followed by good behaviour of health care provider (51%) and access to the facility through mobility aid (45%), as important factors influencing their decision to seek treatment from a heath facility. Participants could select more than one response; therefore the sum of the percentages is greater than 100%.

### Determinants of treatment seeking from formal or informal health care providers

Seeking treatment from formal or informal health care providers was examined with a logistic regression model including all independent variables. The diagnosis of this logistic model is explained in detail in the analysis section of this article. This model helped us to identify the independent variables which have significant statistical explicative power about treatment seeking for general illness by PWPDs. The output of this logistic regression model is presented in Table 2.

**Table 2:** Multivariable logistic regression model predicting primary health care seeking for general illness from formal providers, a cross-sectional study of primary healthcare seeking behaviour of adults with physical disabilities in Bangladesh, November–December 2014

Variables	Odds Ratio	95% Conf. Interval	
Age			
18–30	1.0		
31–45	3.9	(1.2,	13.4)
46–59	13.6	(2.9,	63.7)
60 or over	12.47	(1.7,	93.0)
Sex			
Male	1.0		
Female	4.7	(0.94,	23.6)
Marital status			
Ever married	1.0		
Never Married	3.6	(1.0,	13.8)
Religion			
Muslim	1.0		
Hindu	0.3	(0.1,	0.98)
Educational Attainment			
Less than primary	1.0		
Primary education	0.7	(0.2,	2.1)
Lower secondary education	0.4	(0.1,	1.5)
Upper secondary education	0.6	(0.2,	2.3)
Post-secondary or above	0.6	(0.1,	2.7)
Occupation			
Elementary	1.0		
Government, NGO or private employees	8.7	(1.0,	76.5)
Business	1.3	(0.2,	8.0)
Student	0.7	(0.1,	5.7)
Homemakers	0.1	(0.0,	2.3)
Unemployed	0.2	(0.0,	1.5)
Income			
Lowest	1.0		
Third	0.6	(0.1,	2.6)
Fourth	0.4	(0.1,	1.5)
Highest	0.1	(0.0,	0.8)
Relation to Key earning member in household (HH)			
Self	1.0		
Father or Mother	4.2	(0.9,	199.5)
Brother or Sister	22.8	(0.5,	155.1)
Wife	1.2	(0.2,	5.4)
Others	0.4	(0.0,	22.2)
Educational attainment- key earning member			
Less than primary	1.0		
Primary education	3.2	(1.1,	9.6)
Lower secondary education	1.5	(0.4,	6.1)
Upper secondary education	3.0	(0.8,	11.5)
Post-secondary or above	1.4	(0.2,	7.5)
Occupation-key earning member			
Elementary	1.0		
Government, NGO or private employees	0.6	(0.1,	2.6)
Business	1.2	(0.3,	4.3)
Unemployed	1.4	(0.3,	5.5)
Household income			
Second quintile	1.0		
Lowest	1.5	(0.4,	66.4)
Third	0.6	(0.1	3.0)
Highest	0.2	(0.0,	11.4)
Area of residence			
Urban	1.0	5	
Rural	0.8	(0.4,	1.9)
Type of impairment			
Paraplegia/tetraplegia	1.0		
Hemiplegia	1.9	(0.7,	55.6)
Others	11.2	(0.4,	33.6)
Mobility aid used			
No mobility aid required	1.0		
Wheel chair	4.0	(1.2,	122.9)
Crutch or stick	2.9	(0.8,	66.1)
Last illness suffered			
Fever	1.0		
Urinary tract infection	10.3	(2.3,	46.6)
Sores or ulcer	1313.1	(2.1,	79.3)
Pain	3.6	(1.4,	99.4)
Diarrhea	3.3	(0.9,	12.6)
Others	0.8	(0.3,	2.0)

According to this model, participant’s age, religion, earning member’s education, mobility aid used and last illness suffered were statistically significant to explain seeking treatment from formal health care provider over informal health care provider for their general illness.

The model demonstrated that the older PWPDs were more likely to seek treatment from formal health care provider than younger PWPDs. It indicated that participants who were 31 to 45 years old, 46–59 years old and 60 years old or above, were more likely to seek treatment from formal health care provider than participants who were 18–30 years old (OR = 3.9, [95% CI 1.2 to 13.4]; OR = 13.6, [95% CI 2.9 to 63.7] and OR = 12.5, [95% CI 1.7 to 93.0] respectively). It also evident from this model that the Hindu PWPDs were less likely to seek treatment from formal health care provider than the Muslim PWPDs (OR = 00.3, [95% CI 0.1 to 0.98]). The education level of main earning member was also an important determinant for seeking treatment from formal health care providers. The PWPDs were more likely to seek treatment from formal health care providers when the main earning member had at least a primary education in comparison with those that had less than a primary education (OR = 3.2, [95% CI 1.1 to 9.6]). The model also indicates that the type of mobility aid used was an important predictor of decisions to seek treatment from a formal health care provider. The PWPDs who used wheelchairs were more likely to seek treatment from formal health care providers than those who did not use any mobility aid (OR = 4.0, [95% CI 1.22 to 13]). Moreover, the illness suffered by PWPDs in the past 6 months was another determinant for seeking treatment from a formal health care provider. The PWPDs who suffered from urinary tract infections, sores or ulcers and pain were more likely to seek treatment from formal health care providers than those who suffered from fever (OR = 10.3, [CI 2.3 to 46.6], OR = 13.1, [95% CI 2.11 to 79.3] and OR = 3.6, [95% CI 1.4 to 9.4]). The sex of the PWPDs was marginally significant (OR = 4.7, [95% CI 0.94 to 23.6] to predict the health care seeking behaviour of PWPDs.

## Discussion

This study is the first of its kind in Bangladesh to collect data on primary health care seeking behaviour of PWPDs. A number of studies [14–17] were conducted to understand the health care seeking behaviour of disabled people for the treatment of their disability. However, this study describes the prevalence of PWPDs and identify the determinants of health care seeking behaviours of PWPDs for the treatment of their general illness, apart from their physical disability.

### Factors determining the health care seeking behaviour of PWPDs

This study found that comparatively older individuals (31–45 years, 46–59 years and 60 + years) were more likely to seek treatment from formal health care providers than the younger age group (18–30). Ahmed [19] also demonstrated similar results in his study. He found that older people are more likely to use allopath treatment from formal health care providers than younger people. In his study, the type of illness suffered during the last six months was also a major factor influencing whether participants sought treatment from formal health care providers. The current study demonstrated that participants who suffered from infection, ulcers and pain were more likely to seek treatment from formal health care provider than those who suffered from fever. The explanation could be that these illnesses require or perceived to require more specialized treatment from a formal health care provider in comparison with fever. As a result, PWPDs might visit a formal health care provider rather than an informal health care provider. Moreover, the study demonstrated that the PWPDs who used wheelchairs were more likely to seek treatment from formal health care providers. The explanation could be the same, that PWPDs who used wheelchairs might suffer more from infections and other severe illnesses because of the nature of their physical disability. PWPDs who use a wheelchair may generally require more specialized health care so they might seek treatment from a formal health care provider.

This study identified that fever, pain and infection were the most prevalent general illnesses suffered by the participants over the last six months. This finding is supported by the Bangladesh Household Income and Expenditure Survey [11] which identified ulcer and fever as the two most prevalent illnesses suffered by the general population. Another study [20] in Bangladesh concerning disease patterns and health care seeking behaviour of the general population also observed fever as the most common illness reported by the participants.

### Health care seeking behaviour of PWPDs

The present study demonstrated that 88% of the participants took some form of treatment for their illness and the majority of those participants (69%) took treatment from a formal health care provider. However, other studies [16, 19] have found that the majority of participants preferred to seek treatment from informal health care providers rather than formal health care providers. A study [21] looking at health care seeking behaviour among tribal people in Bangladesh found that participants preferred informal health care providers over formal health care providers because of the high cost associated with seeking treatment from formal health care providers. According to Cockcroft, Milne and Andersson [22], who conducted a study in Bangladesh about health service delivery, people choose informal health care providers over formal health care providers because the cost of visiting an informal health care provider is lower. The possible reasons for visiting formal health care providers by the current study participants might be that they are economically solvent (mean household income of the study participant was around 265 USD), compared to the study conducted by Hossain et al. [16], where around half of the participant’s annual family income was less than 181 USD.

Since most of the participants (60%) were living in urban areas, so it may have been easier for them to seek treatment in government hospitals or non-government organization (NGO) health care facilities where the cost is very low in comparison with private health care providers. This study supports this view because among participants who visited a formal health care provider, only 8% of them visited a private health care provider while 60%, visited a government or NGO health care facilities.

Another important factor was that the participants mostly suffered from infection, pain, and fever. For infection and pain, participants might have felt that they needed treatment from formal health care provider. Among the participants who suffered from infection, all of them sought treatment from formal health care providers. It was also noticeable that when the participants were asked about important factors that influenced their decision to seek treatment from a health care provider, they mentioned good quality treatment and the availability of specialized care as important factors. If quality of treatment and specialized care were perceived to be characteristics of formal health care providers, these factors may have led participants to seek treatment from formal health care providers.

Another finding of this study was that when the participants were from a family which had 6 or more members they were more likely to seek treatment from formal health care providers than those from smaller families. The explanation might be that to see a formal health care provider, the participants needed to travel to a distant place for which they needed support from a family member. Larger families may have facilitated the necessary travel. Moreover, the mean household income of current study participants was around 265 USD. This amount was more than seven times higher than the poverty line set by World Bank [23]. Given their household income, participants in this study may have had adequate resources to seek treatment from formal health care providers rather than going to an informal health care provider.

### Limitations of the study

This study was a descriptive quantitative cross-sectional study. As a result, the current study suffered from several limitations which are discussed below:

1. The main limitation of the study is that data were collected at a specialized health care facility for disabled people. The participants who visited or lived around CRP during the study period were selected for the study. This could lead to selection bias as the timing of data collection could exclude participants who were eligible for the study but were not available during the specific time of data collection. It was also not possible to cover a large number of participants within the short .data collection period. Moreover, the people who visit this specialized health care facility are comparatively wealthy. Therefore, the experiences of these people might be different from those who are living in the community and who do not have the ability to visit a specialized health care facility for their treatment. To address this problem, we selected respondents who have lived in the community at least for 6 months and we retrospectively asked questions about their health care seeking behaviour before visiting CRP. Unfortunately, this cannot completely replicate a study conducted directly in the community.
2. If we had been able to select the participants using random sampling, it would have balanced the baseline characteristics, known and unknown, of the participants. However, this was not possible as there was no comprehensive patient list and the patients who visit the hospital were not fixed. Convenience samples cannot balance the baseline characteristics of the participants and may produce biased results. Moreover, it was not possible to collect data from a large number of people. Initially we calculated the sample size for the study using 95% confidence level and the sample size was 384. However, it was possible to collect data from only 282 participants. To address this problem, we recruited all available participants at that site during the study period; however, we failed to collect data from a representative sample which may have hampered the results.
3. This study was purely a quantitative study; however, we found that to more fully understand the health care seeking behaviour of participants, it was necessary to include some qualitative questions in our questionnaire. We identified that a mixed methods study would be appropriate for this research question. We tried to minimize this problem with literature review and field testing of the questionnaire. Based on the current study, a qualitative study could be conducted to explore certain types of health care seeking behaviours.
4. We collected data retrospectively from the participants to understand their health care seeking behaviour in the community. Some of the participants were living in CRP or in the community around CRP for several months as they received long term treatment for their illness. This might lead to recall bias which could influence the results of the study.

## Conclusions

The study found participant’s age, religion, type of mobility aids used, type of illness suffered, main earning member’s education were associated with primary health care seeking behaviour of PWPDs. As a result, we suggest considering these determinants to devise primary health care interventions for the PWPDs. The study also found that easy access to health care facilities was one of the major factors PWPDs considered when choosing one health care facility over another. It is necessary to think about accessibility issues for PWPDs including building ramps so that the PWPDs can move within the health care facility without help from other people. Moreover, most of the PWPDs in this study visited formal health care providers as they needed specialized care for their general illness other than their disability. Therefore, we propose a primary health care intervention for PWPDs that considers accessibility issues and the need for health care providers who are sensitized to the special needs of PWPDs.

## Abbreviations

BBS: Bangladesh Bureau of Statistics
BDT: Bangladeshi Taka
CRP: Centre for the Rehabilitation of the Paralysed
ERC: Ethical Review Committee
JPGSPH: BRAC James P. Grant School of Public Health
NGO: Non-Governmental Organization
PWPDs: People with physical disabilities
SLP: Summative learning project
WHO: World Health Organization
